# Decoding EEG rhythms offline and online during motor imagery for standing and sitting based on a brain-computer interface

**DOI:** 10.3389/fninf.2022.961089

**Published:** 2022-09-02

**Authors:** Nayid Triana-Guzman, Alvaro D. Orjuela-Cañon, Andres L. Jutinico, Omar Mendoza-Montoya, Javier M. Antelis

**Affiliations:** ^1^Doctorado en Ciencia Aplicada, Universidad Antonio Nariño, Bogota, Colombia; ^2^School of Medicine and Health Sciences, Universidad del Rosario, Bogota, Colombia; ^3^Facultad de Ingeniería Mecánica, Electrónica y Biomédica, Universidad Antonio Nariño, Bogota, Colombia; ^4^Tecnologico de Monterrey, Escuela de Ingeniería y Ciencias, Monterrey, Mexico

**Keywords:** brain-computer interface (BCI), electroencephalogram (EEG), motor imagery (MI), sit-stand, filter bank common spatial pattern (FBCSP), regularized linear discriminant analysis (RLDA), online BCI

## Abstract

Motor imagery (MI)-based brain-computer interface (BCI) systems have shown promising advances for lower limb motor rehabilitation. The purpose of this study was to develop an MI-based BCI for the actions of standing and sitting. Thirty-two healthy subjects participated in the study using 17 active EEG electrodes. We used a combination of the filter bank common spatial pattern (FBCSP) method and the regularized linear discriminant analysis (RLDA) technique for decoding EEG rhythms offline and online during motor imagery for standing and sitting. The offline analysis indicated the classification of motor imagery and idle state provided a mean accuracy of 88.51 ± 1.43% and 85.29 ± 1.83% for the sit-to-stand and stand-to-sit transitions, respectively. The mean accuracies of the sit-to-stand and stand-to-sit online experiments were 94.69 ± 1.29% and 96.56 ± 0.83%, respectively. From these results, we believe that the MI-based BCI may be useful to future brain-controlled standing systems.

## 1. Introduction

A brain-computer interface (BCI) system provides a communication channel between the brain and an external device. These systems have been developed for decades and the choice of the BCI paradigm depends on the application (Lotte et al., 2018b). Among the possible strategies reported in the literature, the most successful noninvasive BCI paradigms are based on three main approaches: evoked response (P300), steady-state visually evoked potential (SSVEP), and motor imagery (MI) (Lee et al., 2019). However, research on electroencephalogram (EEG) based MI of lower limb movements toward BCI-controlled applications remains relatively scarce (Bobrova et al., 2020; Asanza et al., 2022). Many of these studies have only been tested in offline scenarios due to the complexity of the movements and experimental setups that produce unrealistic EEG signals when compared to experimental setups in online scenarios (Rodríguez-Ugarte et al., 2017). Only a small number of studies have been conducted on standing and sitting behaviors in offline scenarios (Zhou et al., 2007; Bulea et al., 2014; Singh et al., 2017; Chaisaen et al., 2020). Therefore, we examined the use of an EEG-based BCI to decode offline and online MI information for these types of movements.

In recent decades, a wide variety of methods have been developed to decode motor imagery tasks from EEG signals in order to improve the performance of BCI systems (George et al., 2021; Singh et al., 2021). These methods include feature extraction techniques that use temporal (Rodríguez-Bermúdez and García-Laencina, 2012; Hamedi et al., 2014; Kee et al., 2017; Samuel et al., 2017), spectral (Al-Fahoum and Al-Fraihat, 2014; Oikonomou et al., 2017), and time-frequency representations (Kevric and Subasi, 2017; Gao et al., 2018; Aggarwal and Chugh, 2019; Padfield et al., 2019; Ortiz et al., 2020). Nevertheless, the usefulness of spatial filtering techniques in BCI applications has been explored for many years now, as a way to select the most discriminative features in EEG recordings for motor imagery tasks, as well as to reduce the huge dimensionality that can be present in feature spaces (Ang et al., 2012; Congedo et al., 2017; Lotte et al., 2018a; Rejer and Górski, 2018). In this sense, the common spatial pattern (CSP) method has been shown to extract discriminative information more effectively than other spatial filters such as bipolar, Laplacian, or common average reference, as well as unsupervised data-driven techniques such as independent component analysis (ICA) (Naeem et al., 2009; Ortner et al., 2015; He and Wu, 2018). While research already exists concerning the CSP method and was successfully applied (Chaisaen et al., 2020), important knowledge is still missing regarding the challenge of decoding EEG rhythms online during motor imagery tasks for standing and sitting.

Some researchers use the term “BCI illiteracy” for people unable to control a BCI (Allison and Neuper, 2010; Ahn et al., 2013). Nevertheless, the effective control threshold depends on many factors, including the BCI application and paradigm (Edlinger et al., 2015; Lee et al., 2019). For example, an accuracy level of less than 80% might be insufficient in a BCI system designed for communication. Conversely, for BCI systems intended for motor rehabilitation purposes, an accuracy above the confidence level might become sufficient (Thompson, 2018). Previous studies have focused on decoding EEG signals of left-hand and right-hand motor imagery tasks (which represented sitting down and standing up) (Noda et al., 2012; Wang et al., 2018), or SSVEP signals (in which flickering lights corresponded to the command for standing and sitting) (Kwak et al., 2017), instead of investigating the decoding of continuous EEG rhythms during motor imagery concerning standing and sitting. For these reasons, considering the applications of lower limb motor imagery and the necessity of using an online BCI for these little-researched movements, this study establishes an offline and online performance analysis of an EEG-based BCI during motor imagery tasks for standing and sitting.

In the present study, we investigated whether people could control an EEG-based BCI using motor imagery for standing and sitting movements. For this purpose, we explored two different classification scenarios: offline and online. The goal of the offline scenario was to obtain individual training sets for each participant in offline experiments to adjust and evaluate the machine learning models of the BCI (one model for sit-to-stand, one model for stand-to-sit). After training the interface, the online scenario aimed to measure the speed and accuracy of the BCI to decode EEG rhythms in real time during motor imagery tasks for standing and sitting. To our knowledge, the proposed EEG-based BCI is the first one to recognize motor imagery tasks online for standing and sitting, which is crucial for implementing brain-controlled standing technology. The filter bank common spatial pattern (FBCSP) method was used for feature extraction based on the modulation of theta wave (4–8 Hz) and sensorimotor rhythm (SMR), which includes two bands in the spectrum: alpha (8–12 Hz) and beta (12–30 Hz), which are associated with movement-related tasks in physical activity execution, motor planning, intention to move, and motor imagery (Yuan and He, 2014). To make the classification as fast and simple as possible, the regularized version of the linear discriminant analysis (RLDA) approach was used.

## 2. Materials and methods

### 2.1. Participants

The study involved 32 healthy subjects aged 19–29 years (16 women and 16 men). The mean (± standard deviation) age of the participants was 22.4 (± 2.3) years. None of the participants reported a history of neurological, musculoskeletal, or other disorders, and all had normal or corrected-to-normal vision. All participants were undergraduate students, with no academic relationship to the experimenters, and none had previous experience with EEG or BCI experiments. Before starting their experimental session, participants were duly informed of the nature of the study and instructed on the correct execution of the experiments. In addition, participants voluntarily signed an informed consent form in accordance with the experimental protocol approved by the ethics committee of the Universidad Antonio Nariño. This experimental protocol followed the standards of the Declaration of Helsinki (Association, 2013). Each subject was paid for their participation at the end of their session.

### 2.2. Electroencephalographic data recording

**Figure 1:**
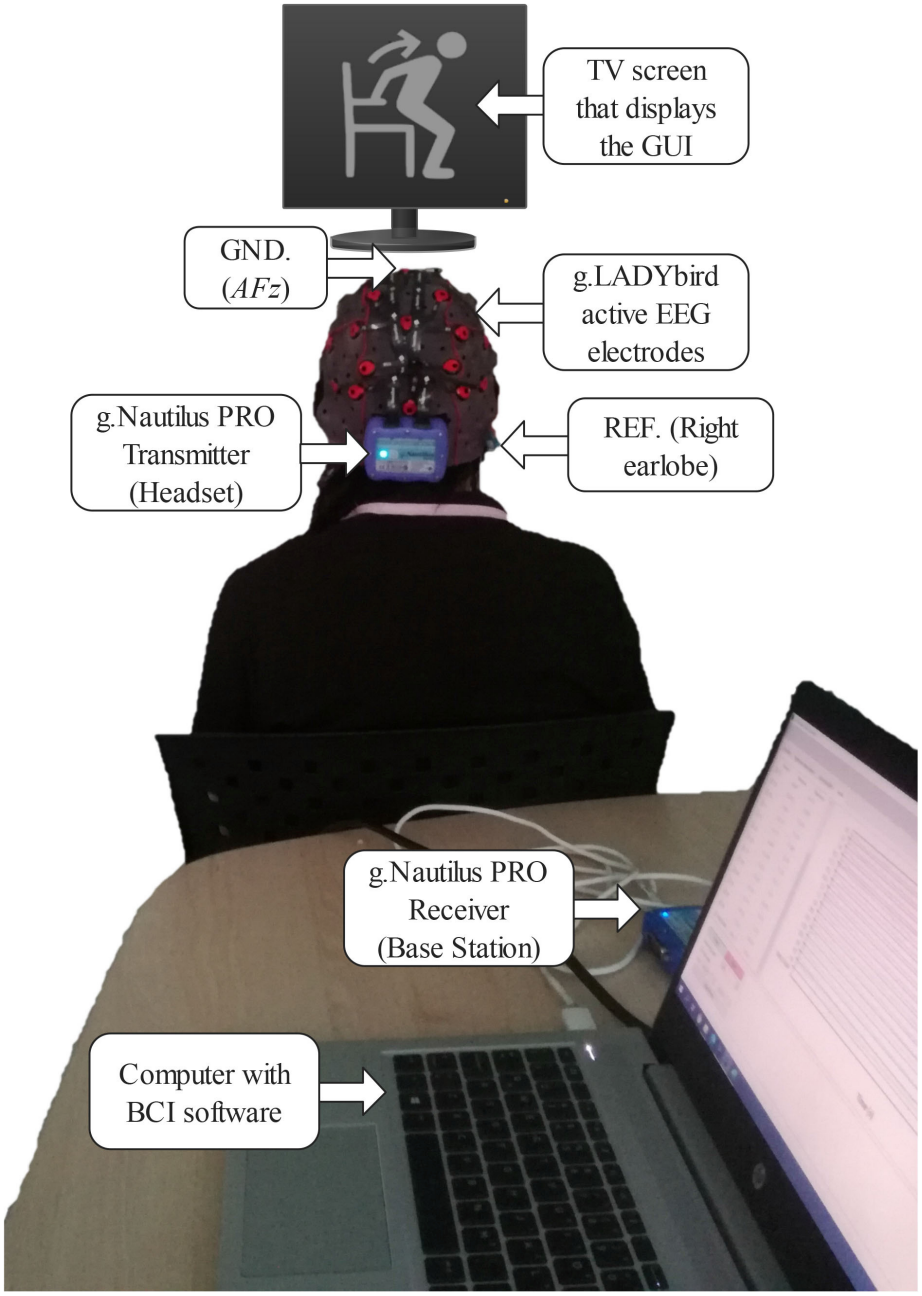
Experimental setup with a participant in front of the TV screen with the graphical user interface (GUI) to present visual cues regarding each step in the sequence of a trial. The participant is equipped with a g.Nautilus PRO system with 17 gel-based active electrodes (g.LADYbird technology) to acquire the electroencephalogram (EEG) and transmit it wirelessly from the headset (transmitter) to the receiver (base station) connected to the computer with the BCI software. REF, reference electrode; GND, ground electrode at *AFz*.

EEG data were obtained from 17 active wet electrodes (g.LADYbird) mounted on a g.Nautilus PRO biopotential amplification system (g.tec medical engineering GmbH, Austria) with wireless data transmission technology (see Figure 1). Electrodes were moistened with conductive gel and placed according to the international 10–20 system at the following positions around the primary motor cortex (Xu et al., 2017): *F*3, *Fz*, *F*4, *FC*5, *FC*1, *FC*2, *FC*6, *C*3, *Cz*, *C*4, *CP*5, *CP*1, *CP*2, *CP*6, *P*3, *Pz* and *P*4, with the ground (GND) electrode placed at *AFz* and the reference (REF) electrode placed in the right earlobe. EEG signals were acquired at a sampling rate of 250 Hz and digitally band-pass filtered with cutoff frequencies from 0.01 Hz to 60 Hz, using 6*th* order Butterworth filter at each electrode (Podder et al., 2014). Before starting the EEG recording, the impedance of the electrodes was verified to be below 30 kΩ using the impedance measurement tool provided by the manufacturer of the g.Nautilus PRO. Additionally, an in-house software platform developed in C++ was used to manage and control the execution of the experiment, collect EEG signals, store the data, and process them both offline and online (Copyright @ 2018 Instituto Tecnologico y de Estudios Superiores de Monterrey).

### 2.3. Experimental design

The experiments were conducted in an acoustically isolated room where only the participant and the experimenter were present. The participant was seated in a chair in a posture that was comfortable for him/her but did not affect data collection. In front of the participant, a 40-inch TV screen was placed at about 3 m, as shown in Figure 1. On this screen, a graphical user interface (GUI) displayed images that guided the participant through the experiment. Each experimental session was divided into two phases: an offline phase and an online phase.

#### 2.3.1. Offline phase

**Figure 2:**
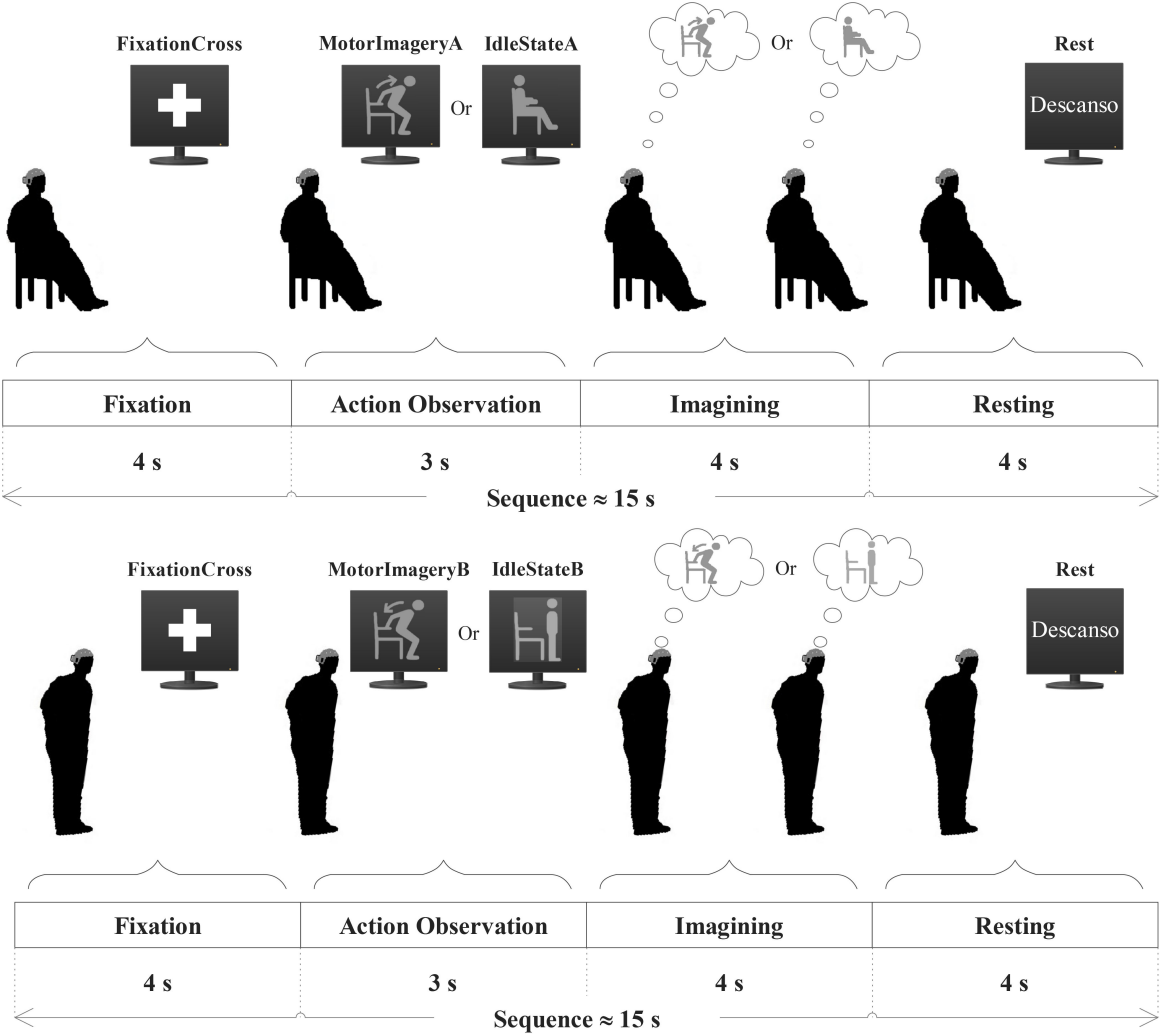
Illustration of the temporal sequence of a trial performed by the participant in the sit-to-stand experiment **(Top)** and in the stand-to-sit experiment **(Bottom)**. Each sequence consisted of four steps: fixation, action observation, imagining, and resting.

The offline experiments consisted of recording participants' EEG signals during motor imagery trials for standing and sitting that were guided by the GUI presented on the TV screen (see Figure 2). Just before starting the recording of the EEG signals, the participants practiced the sequences of mental tasks that were indicated by the GUI on the TV screen. Once the recording of the EEG signals started, six offline runs were conducted in which the participants were standing in three runs and sitting in the other three runs. The participant could choose the order of the runs, and between each run, there was a break of a few minutes for the participant to avoid fatigue and boredom, recover, and prepare to continue with the recording of the next run.

In each run, the participant had to repeat a block of 30 trials of mental tasks indicated by visual cues continuously presented on the screen in a pseudo-random sequence. The temporal sequence of mental tasks performed by each participant is shown in Figure 2. Each sequence of a trial consisted of four steps:

1. Fixation: As a first step, a cross symbol appeared on the TV screen for 4 s during which the participant was asked to avoid any body movement or effort and to stay focused while looking at the symbol.
2. Action observation: In the second step, a figure appeared on the TV screen for 3 s, which the participant had to observe and perform one experimental task subsequently in the third step.
3. Imagining: In the third step, the participant had to visualize the action indicated by the figure shown in the second step and perform one experimental task for 4 s in response to the figure. For instance, the task could be sitting motionless while actively imagining the sit-to-stand movement (labeled as MotorImageryA), sitting motionless without imagining the sit-to-stand movement (labeled as IdleStateA), standing motionless while actively imagining the stand-to-sit movement (labeled as MotorImageryB), or standing motionless without imagining the stand-to-sit movement (labeled as IdleStateB).
4. Resting: Finally, in the fourth step of the sequence, the text “Descanso” (the Spanish word for “Rest”) appeared on the TV screen for 4 s, instructing the participant to rest from the experimental task, blink, or move the head and body if necessary.

Participants were asked to avoid or minimize muscle effort and blinking from the first step to the third step of each sequence. For each participant, an offline experimental session was conducted for the construction of two datasets: (*A*) Sit-to-stand and (*B*) Stand-to-sit. The participant's EEG data were collected from 90 sequences of dataset *A* (45 trials of MotorImageryA tasks and 45 trials of IdleStateA tasks) and 90 sequences of dataset *B* (45 trials of MotorImageryB tasks and 45 trials of IdleStateB tasks). In total, the time duration of the offline experimental session was at least 1 h for the collection of the two EEG datasets.

##### 2.3.1.1. EEG data preprocessing

The two EEG datasets recorded from the offline experiments were independently subjected to the following automatic pre-processing pipeline (Mendoza-Montoya, 2017). First, the EEG data were split into epochs (2-s data segments of contiguous sample points of each trial) from 1 s before to 1 s after beginning the experimental task of step 3 in the sequence of a trial. Second, each epoch was separated and labeled according to one of the four experimental tasks: MotorImageryA, IdleStateA, MotorImageryB, or IdleStateB, and *E*_*k*_ ⊆ {1, 2, …, 45} was the subset of indices of the epochs that belong to the task *k* ∈ {MotorImageryA, IdleStateA, MotorImageryB, IdleStateB}. For each participant, the total number of epochs for each pair of experimental tasks was *n*_epochs_ = 90. Third, each EEG epoch *X* = [*x*_*e*_(*t*)]*n*_*e*_×*n*_*t*_ of *n*_*e*_ electrodes (or 17 EEG channels) and *n*_*t*_ sample points (500 sample points per EEG epoch) was filtered using digital finite impulse response (FIR) filters with cut-off frequencies between 20–40 and 4–40 Hz. The result of this filtering step was the signal $X^{20-40}\left(t\right)=\left[x_{e}^{20-40}\left(t\right)\right]\in {\mathbb{R}}^{n_{e}\times n_{t}}$ to detect muscle artifacts and the signal $X^{4-40}\left(t\right)=\left[x_{e}^{4-40}\left(t\right)\right]\in {\mathbb{R}}^{n_{e}\times n_{t}}$ to encompass the motor-related frequency bands of the oscillatory EEG activity. Then, the peak-to-peak voltage $V_{e}^{\text{pp}}$, the standard deviation σ_*e*_, and the normalized power $P_{e}^{\text{norm}}$ of each channel were calculated as below:


(1) $$\begin{array}{l} V_{e}^{\text{pp}}=max_{t}\left(x_{e}^{4-40}\left(t\right)\right)-min_{t}\left(x_{e}^{4-40}\left(t\right)\right), \end{array}$$

(2) $$\begin{array}{l} \sigma_{e}=\sqrt{\frac{1}{n_{t}-1}\overset{n_{t}}{\underset{t=1}{\sum }}{\left(x_{e}^{4-40}\left(t\right)-\mu_{e}\right)}^{2}}, \end{array}$$

(3) $$\begin{array}{l} P_{e}^{\text{norm}}=\frac{\overset{n_{t}}{\underset{t=1}{\sum }}{\left(x_{e}^{20-40}\left(t\right)\right)}^{2}}{\overset{n_{t}}{\underset{t=1}{\sum }}{\left(x_{e}^{4-40}\left(t\right)\right)}^{2}}, \end{array}$$

where


(4) $$\begin{array}{l} \mu_{e}=\frac{1}{n_{t}}\overset{n_{t}}{\underset{t=1}{\sum }}x_{e}^{4-40}\left(t\right). \end{array}$$

The processed data for each experimental task from each participant contained a collection of epochs × time points × channels (45 × 500 × 17). The following exclusion criteria were applied to identify and discard noisy epochs: (*i*) Maximum peak-to-peak value $V_{e}^{\text{pp}}$ greater than 200 μV; (*ii*) Standard deviation amplitude σ_*e*_ greater than 50 μV; and (*iii*) Noise to signal ratio $P_{e}^{\text{norm}}$ greater than 0.7. These criteria may indicate if the subject is blinking, the amplifier is saturated, the electrodes are not making good contact with the scalp, or there are some muscle artifacts, as suggested in Mendoza-Montoya (2017), Delijorge et al. (2020), and Hernandez-Rojas et al. (2022). Finally, any epoch where at least one electrode met these criteria was visually inspected to rule out noise-contaminated trials (as a double check) and labeled as an “artifact” manually. The trials with epochs labeled as “artifacts” were discarded and were not used in the subsequent analysis. Conversely, the epochs below the threshold levels passed validation and were used to investigate spatially discriminative EEG features with the filter bank common spatial pattern (FBCSP) method.

##### 2.3.1.2. EEG signals analysis

Time-frequency analysis (TFA) of EEG time series is a suitable technique to study cognitive events, such as motor imagery tasks, that induce transient power modulations of the EEG spectrum (Graimann and Pfurtscheller, 2006; Zhang, 2019). Modulations of this kind appear as a decrease (event-related desynchronization or ERD) or an increase (event-related synchronization or ERS) of spectral power at specific frequency bands (Pfurtscheller and Lopes da Silva, 1999). ERD/ERS is also known as an event-related spectral perturbation (ERSP), which measures the event-related spectral changes relative to a reference interval used as the spontaneous EEG baseline in a wide range of frequencies (Makeig, 1993). Therefore, TFA was performed on the aforementioned preprocessed trials to visualize the ERD/ERS patterns using the EEGLAB toolbox (version 2021.1) (Delorme and Makeig, 2004).

The resting stage of every trial was discarded and not considered in the present study, as it does not contain relevant EEG activity for the analysis. ERSP was computed at the frequency ranges from 4 to 30 Hz for all channels to calculate the power spectrum by applying the Morlet wavelets transform with incremental cycles (7 cycles at the lowest frequency to 14 at the highest), resulting in 200-time points (−6.03, 3.02) s. The baseline reference was then taken from −3.5 to −3 s (which corresponds to the non-movement interval) at the beginning of step 3 in the sequence of each trial. Spectral power changes were averaged at each time point and normalized by baseline spectra. The significance of ERSP deviations from the baseline was analyzed using the bootstrap method (α = 0.05) (Graimann and Pfurtscheller, 2006). Accordingly, ERSP could identify significant ERD and ERS as negative and positive spectral changes, respectively (Zhang, 2019).

##### 2.3.1.3. Feature extraction

One of the most successful algorithms in BCI research for feature extraction is the common spatial pattern (CSP) (Padfield et al., 2019). This method finds spatial filters that project EEG data into a new space in which the variances corresponding to one class are maximized while the variances of a second class are minimized (Lotte and Guan, 2011). In this study, an enhanced version of the original CSP algorithm, known as the filter bank common spatial pattern (FBCSP) algorithm (Ang et al., 2012), was implemented using a FIR filter bank of five digital band-pass filters centered on five EEG frequency bands (theta: 4–8 Hz, alpha: 8–12 Hz, low-beta: 12–16 Hz, mid-beta: 16–20 Hz, high-beta: 20–30 Hz) (Chen et al., 2018). All the filters in the filter bank were designed in the frequency domain using a Gaussian kernel with unitary gain.

The FBCSP algorithm is useful when the frequency components of the modulated signals may vary among subjects. For instance, in the motor imagery paradigm, a particular frequency of the sensorimotor rhythm is not the same for all users (Saha and Baumert, 2020). For this reason, each preprocessed epoch of a training set was filtered using the FIR filter bank in order to obtain spectrally filtered epochs *Y* = [*y*_*e, f*_(*t*)]*n*_*e*_×*n*_*t*_×*n*_*f*_, where *n*_*f*_ is the EEG frequency subband (4–8 Hz, 8–12 Hz, 12–16 Hz, 16–20 Hz, 20–30 Hz).

We studied the bi-class classification of two pairs of experimental tasks: MotorImageryA vs. IdleStateA for the sit-to-stand transition and MotorImageryB vs. IdleStateB for the stand-to-sit transition. The CSP algorithm was then applied to each subband and each pair of the experimental tasks. The CSP algorithm provided an *n*_*e*_ × *n*_*e*_ projection matrix $W={\left[w_{1},w_{2},\ldots ,w_{n_{e}}\right]}^{{\;}^{\prime }}$ for each pair of experimental tasks. This matrix was a set of subject-dependent spatial patterns, reflecting the specific activation of cortical areas during the experimental task. With the projection matrix *W*, the decomposition of an epoch *Y* was described by *Z* = *WY*, where this transformation projected the variance of the filtered EEG signals of *Y* onto the rows of *Z* and gave rise to *n*_*e*_ new time series. The columns of *W*^−1^ were a set of CSPs that can be thought of as time-invariant EEG source distributions (Ortner et al., 2015).

The number of spatial filters retained was chosen as six for all subjects and training sets, as recommended in Blankertz et al. (2008b). The three first spatial filters contribute most to the variance of class one data, and the last three spatial filters contribute most to the variance of class two data. If *n*_*m*_ = 6 represents the number of spatial filters retained per frequency subband, the spectrally filtered epochs *Y* are transformed into spatially filtered epochs $Y^{\text{CSP}}={\left[y_{e,f}^{\text{CSP}}\left(t\right)\right]}_{n_{m}\times n_{w}\times n_{t}\times n_{f}}$, where *n*_*w*_ is the number of time windows of *n*_*t*_ time points for each EEG epoch. During the offline feature extraction, each EEG epoch was split into two-time windows of intervals [−1, 0) s and [0, 1) s, respectively, where 0 s is the onset time of step 3 in the sequence of a trial. Finally, for each frequency subband and projected channel, the BCI calculated the log-variance. This resulted in 30 features (*n*_*m*_ × *n*_*f*_) by 180 observations (*n*_epochs_ × *n*_*w*_) for each pair of experimental tasks from each participant that would be used in the regularized linear discriminant analysis (RLDA) classifier. The Fisher's criterion was applied to evaluate the extracted features.

##### 2.3.1.4. Classifier

Linear classifiers have proven to be an efficient option for the detection of EEG rhythms in motor imagery paradigms for BCI applications (Oikonomou et al., 2017). In this category, linear discriminant analysis (LDA) can provide optimal results and outperform more complex classification techniques. Additionally, LDA is relatively easy to train and evaluate and requires a low computational cost to classify new observations. Therefore, two binary classification models based on LDA with regularized covariances were used to discriminate with a first model, MotorImageryA vs. IdleStateA for the sit-to-stand classification scenario, and with a second model, MotorImageryB vs. IdleStateB for the stand-to-sit classification scenario.

These types of binary-class models are highly employed in MI-based BCI applications (Lotte et al., 2018a). The proposed BCI employed the regularized linear discriminant analysis (RLDA) as a classification machine learning model to decide what class to assign to the processed data according to a linear combination of the feature vector (Fu et al., 2019). If *x* represents a real vector of *n*_*c*_ = 30 features for an EEG epoch, the classification model evaluates the function


(5) $$\begin{array}{l} f\left(x\right)=g\left(\overset{n_{c}}{\underset{i=1}{\sum }}b_{i}x_{i}+d\right), \end{array}$$

where $b={\left[b_{1},b_{2},\ldots ,b_{n_{c}}\right]}^{{\;}^{\prime }}$ and *d* are the coefficients of the linear model, and *g*(*a*) is a scalar function. Then, the classification model returns a label or category *l* ∈ {1, −1} to the given observation based on the evaluation of *f*(*x*). A typical approach is to use a threshold value such that values above it have the class label *l* = 1. Conversely, values below this threshold correspond to the other class label *l* = −1.

LDA finds the class label *l* that maximizes the conditional probability *p*(*L* = *l*|*X* = *x*) (Ng and Jordan, 2001). It assumes that the probability density functions *p*(*X* = *x*|*L* = −1) and *p*(*X* = *x*|*L* = 1) are both normally distributed with mean vectors *m*_−1_, *m*_1_ and covariance matrices *C*_−1_, *C*_1_. Under these assumptions, the decision rule *p*(*L* = 1|*X* = *x*) > *p*(*L* = − 1|*X* = *x*) is expressed as a dot product *b*′*x* + *d* > 0, where


(6) $$\begin{array}{l} b=2C^{-1}\left(m_{1}-m_{-1}\right), \end{array}$$

(7) $$\begin{array}{l} d=\ln\left(\frac{P(L=-1)}{P(L=1)}\right)+{m^{\prime }}_{\;-1}C_{-1}^{-1}m_{-1}-m^{\prime }{\;}_{1}C_{1}^{-1}m_{1}, \end{array}$$

and *P*(*L* = *l*) is the probability of class label *l*. Additionally, for the automatic regularization of the LDA algorithm, the BCI uses the method proposed by Ledoit and Wolf (2004) to compute *C*_−1_ and *C*_1_ (Lotte and Guan, 2011).

The goal of the two classification models was the discrimination of the different pairs of experimental tasks to return a motor imagery state or class label *l* that represented when the participant was imagining (*l* = 1) or not imagining (*l* = −1) a movement based on the observations or corresponding resulting features for each EEG epoch. For this purpose, the BCI incorporated two RLDA classifiers to complete the two machine learning models for the sit-to-stand and stand-to-sit transitions, respectively. These classifiers are simple and have a low computational requirement, which makes them suitable for the online BCI (Mao et al., 2017).

##### 2.3.1.5. Classification model performance

In the final steps of the offline phase, the two complete machine learning models of the study subject were evaluated for the transitioning actions: (*i*) Sit-to-stand and (*ii*) Stand-to-sit. The evaluation of these models depends on the collection of labeled data obtained from datasets *A* and *B* in the offline experiments. For this assessment, the machine learning models *i* and *ii* for each participant were independently assessed by applying a five-fold cross-validation procedure to avoid overfitting and measure generalization on each model (Berrar, 2019). In this procedure, the set of trials of *A* and *B* were randomly split into five equal-sized subsets each, respectively. For each fold, the BCI uses four subsets to train the models *m*∈{*i, ii*}. Then, the remaining subset is used to test the corresponding model *m*. This process was repeated with mutually exclusive training and test subsets until the five cross-validations were completed. The classification accuracy *acc*_*m, c*_ of each class *c*∈{MotorImagery, IdleState} was calculated as described below:


(8) $$\begin{array}{l} acc_{m,c}=\frac{n_{\text{correct}}}{n_{\text{total}}}\times 100\%, \end{array}$$

where *acc*_*m,c*_ is the offline accuracy, *n*_total_ is the total number of instances of class *c*, and *n*_correct_ is the number of instances classified correctly in class *c* by model *m*. The overall model accuracy *acc*_*m*, overall_ = 0.5 × (*acc*_*m*,MotorImagery_+*acc*_*m*,IdleState_) and the confusion matrices were also computed.

Additionally, permutation testing was applied to assess the significance level of the overall model accuracies (Good, 2006). This test repeats the five-fold cross-validation procedure by shuffling the class labels during the training of the classifiers to compute the empirical random classification accuracy. In this methodology, the null hypothesis (*H*_0_) indicates that observations of both classes are exchangeable so that any random permutation of the class labels produces similar accuracies to the obtained with the non-permuted data. The alternative hypothesis (*H*_1_) is accepted when the overall model accuracy is an extreme value in the empirical distribution built with several random permutations of the labels. When the alternative hypothesis is accepted, we can say that the overall model accuracy is above the chance level.

#### 2.3.2. Online phase

For each participant, the two machine learning models obtained in the offline phase were used to carry out two online experiments: (*I*) Sit-to-stand and (*II*) Stand-to-sit. Each participant was instructed to select, in no particular order, 30 sequences for experiment *I* (15 trials of MotorImageryA tasks and 15 trials of IdleStateA tasks) and 30 other sequences for experiment *II* (15 trials of MotorImageryB tasks and 15 trials of IdleStateB tasks). Each trial was unique and was generated pseudo-randomly before the experiment.

The timeline of the online sequences was indicated by the user interface in the same way as shown in Figure 2. However, the difference between the offline and online timelines was in step 3. In the third step of the online timeline, the participant performed one experimental task in response to the figure shown in step 2, and the BCI attempted to detect this task in real time for 3–15 s at the same time that provided feedback.

Technically, the online classification of single-trial EEG data could be done as in the offline phase since the trained classifiers can be applied to feature vectors calculated from an arbitrary window. However, this is likely to lead to unreliable results since those classifiers are adjusted to detect signals with a specific time related to the response (Blankertz et al., 2001). There is no guarantee that the classifier will behave similarly elsewhere. As suggested in Blankertz et al. (2001, 2008a), Syan and Harnarinesingh (2010), and Mendoza-Montoya (2017), sliding windows are usually used to increase online classification's robustness to time-shifted signals. Thus, during the online feature extraction, the data length of each epoch was 1 s, that was 250 sample points, and the BCI split them into five sliding windows (50 sample points each), resulting in five feature vectors from each trial.

**Figure 3:**
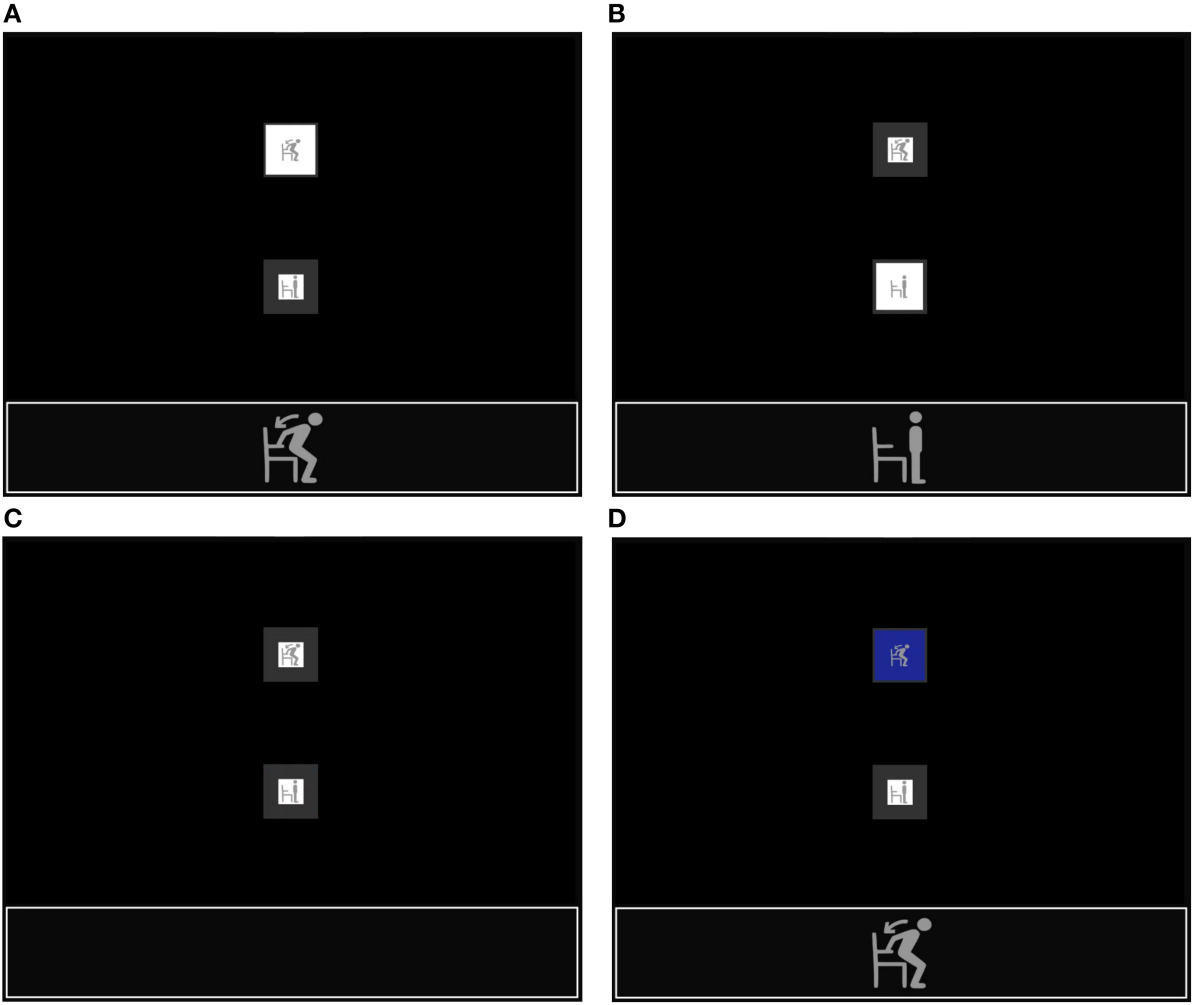
Continuous visual feedback during the online stand-to-sit experiment when: **(A)** the BCI has detected a high MI-related activity, **(B)** the BCI has detected a high idle state-related activity, **(C)** the BCI has not detected a dominant experimental task, and **(D)** the BCI has detected a dominant experimental task.

The BCI processed and provided continuous visual feedback on the results obtained after classifying five consecutive time windows (50 sample points each) from one epoch (250 sample points). If one experimental task produces high MI-related activity, the BCI makes the white background of the corresponding figure look bigger (see Figure 3A). If the idle state-related activity is higher, that figure's white background looks bigger (see Figure 3B). Otherwise, both figures' backgrounds are the same size (see Figure 3C). This visual feedback notifies the participant when the BCI is detecting MI-related activity and helps to increase the MI modulations of the intended movement (Yu et al., 2015).

**Figure 4:**
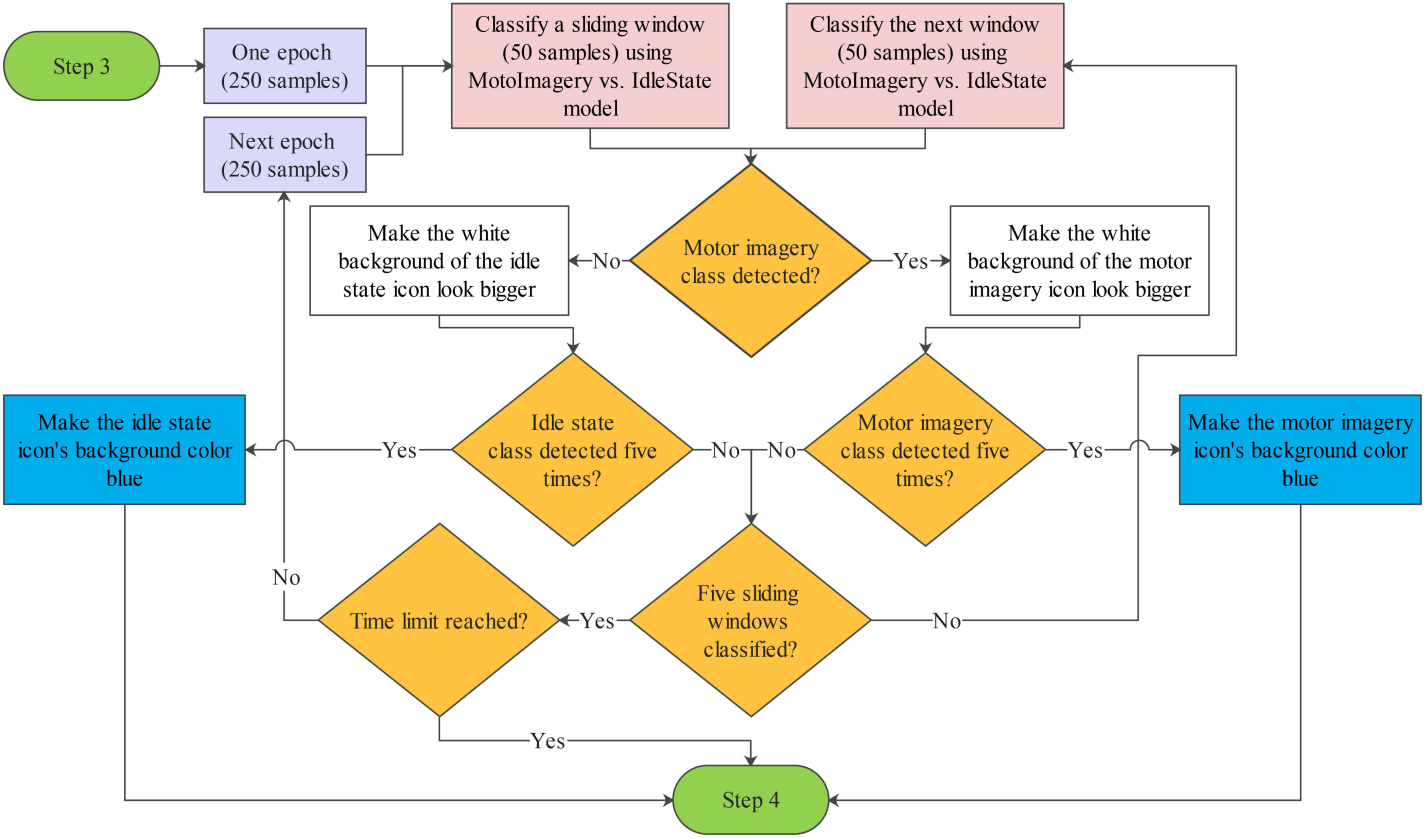
Flowchart of the online classification. In online conditions, the BCI processes each time window of the epoch and assigns it one class between motor imagery and idle state. The BCI also provides continuous visual feedback on the classifications by making the white background of the icon corresponding to the class look bigger or turn blue. The time limit to detect five consecutive times the same class and pass step 3 is 15 s.

As shown in Figure 4, the RLDA classifier labeled each of the time windows from 50 sample points with the name of one of the experimental tasks. The label might be used directly to determine the action or control command to produce with the BCI. However, because the accuracy of the MI-based BCI is typically below 90%, the risk of executing the wrong action is high (Irimia et al., 2018). For this reason, the BCI only generates command or action signals when an experimental task has been detected several times for a few seconds. The minimum time required is about 3 s: 1 s to acquire a whole epoch of EEG signals, another second to classify the EEG data five times, and a third second to select a command or action to execute.

When the same experimental task label has been detected five consecutive times in one epoch, the BCI synchronizes the state of the GUI to produce visual feedback on the selected task, and the corresponding figure background is colored blue (see Figure 3D). On the other hand, if the BCI does not detect the same label five consecutive times, it waits for new labels and dismisses the oldest ones. If the BCI detects the requested experimental task in less than 15 s, the sequence is interrupted to provide visual feedback (see Figure 3D) and continues with step 4 (see Figure 4). On the contrary, if the BCI does not recognize the experimental task and reaches the time limit of 15 s, the BCI simply continues with step 4.

##### 2.3.2.1. Online BCI evaluation

The online assessment aims to investigate the feasibility of decoding in real time the two classes (MotorImagery vs. IdleState) of the two binary machine learning models in the sit-to-stand and stand-to-sit experiments. Consequently, the online evaluation procedure was carried out for each participant independently. Two machine learning models were used for each participant to assess the feasibility of continuous detection of motor information along the online trials. To this end, the performance of the BCI was evaluated in terms of the following detection metrics that were calculated using Equations (9)–(13).


(9) $$\begin{array}{l} TPR=\frac{TP}{TP+FN}, \end{array}$$

(10) $$\begin{array}{l} TNR=\frac{TN}{TN+FP}, \end{array}$$

(11) $$\begin{array}{l} acc_{\text{online}}=\frac{TP+TN}{TP+TN+FP+FN}, \end{array}$$

(12) $$\begin{array}{l} PPV=\frac{TP}{TP+FP}, \end{array}$$

(13) $$\begin{array}{l} NPV=\frac{TN}{TN+FN}, \end{array}$$

where:

- Sensitivity or true positive rate (*TPR*) indicates the percentage of times that the motor imagery class was detected correctly (*TP* are the true positives and *FN* are the false negatives).
- Specificity or true negative rate (*TNR*) denotes the percentage of times that the idle state class was detected correctly (*TN* are the true negatives and *FP* are the false positives).
- Accuracy (*acc*_online_) represents the probability of correctly detecting the motor imagery and idle state classes given the total number of attempts to detect them.
- The positive predictive value (*PPV*), also called precision, is the probability that the detection of the motor imagery class is correct given the total number of times that class is detected.
- The negative predictive value (*NPV*) is the probability that the detection of the idle state class is correct given the total number of times that class is detected.

The information transfer rate (*ITR*) was also used as a performance metric for the online evaluation of the BCI. The calculation of this metric is based on the amount of information transferred per unit of time. The *ITR* was calculated for each participant in bits/min using the following formula (He et al., 2018):


(14) $$\begin{array}{l} ITR=\frac{60}{T}\times [1+(acc_{\text{online}})\log_{2}(acc_{\text{online}})\\ \;+(1-acc_{\text{online}})\log_{2}(1-acc_{\text{online}})], \end{array}$$

where T is the average time from task performing to task detection (detection time in seconds). Under these conditions, the maximum possible information transfer rate is 20 bits/min for each online experiment (Wolpaw et al., 2002).

## 3. Results

**Figure 5:**
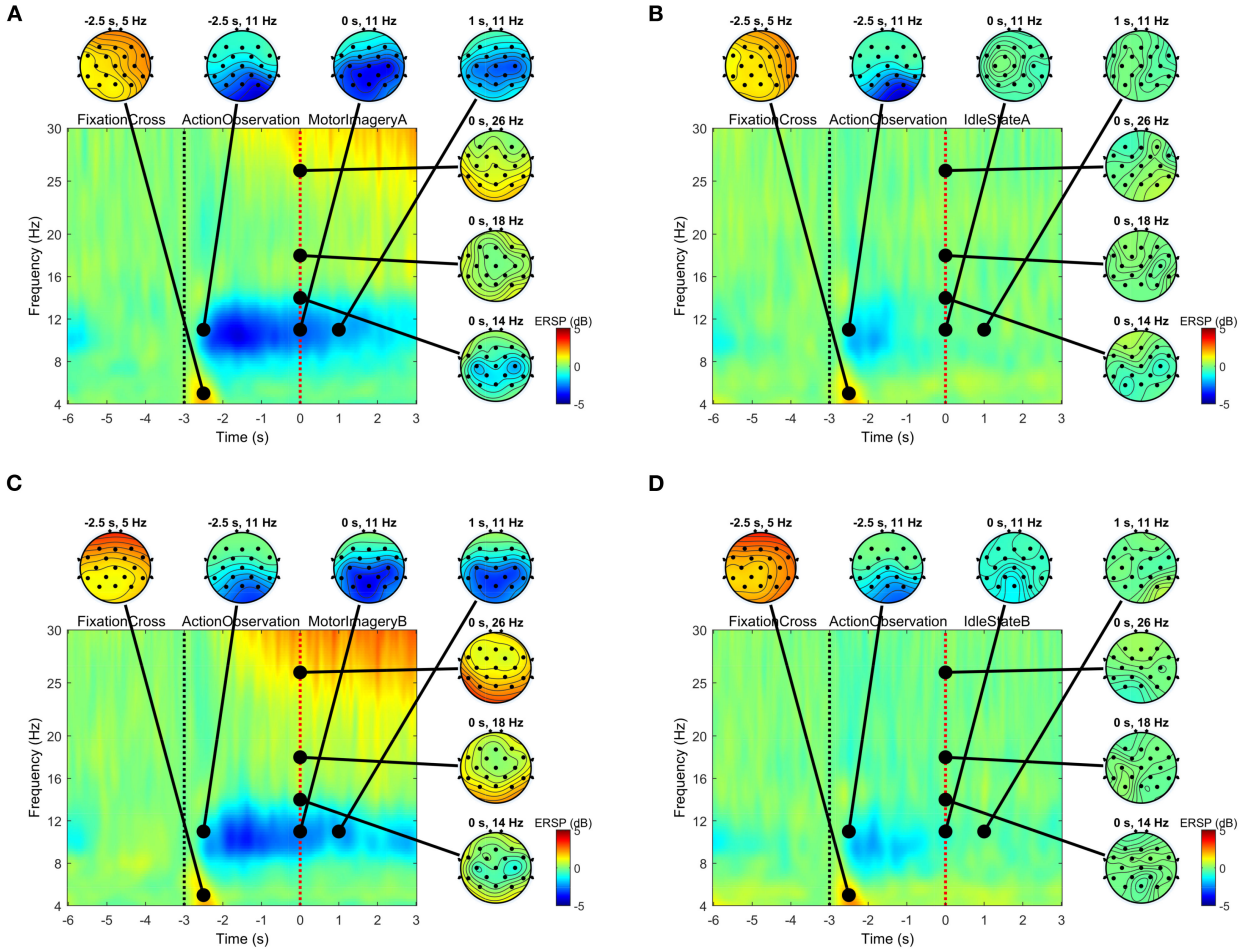
Group-level event-related spectral perturbation (ERSP) for frequencies between 4 and 30 Hz across all trials pooled for sit-to-stand (top panels) and stand-to-sit (bottom panels) experiments compared to the baseline from −3.5 to −3 s. Time-frequency (TF) plots combined the TF decompositions across all channels. Note that these plots with the 2-D scalp topographies also combined ERSP from different subjects and all present ERSP values were statistically significant compared to the baseline (α = 0.05). The time interval (−6, −3] s corresponds to the fixation stage, (−3, 0) s corresponds to the action observation stage, and [0, 3) s corresponds to the stage of one of the four experimental tasks: **(A)** MotorImageryA, **(B)** IdleStateA, **(C)** MotorImageryB, or **(D)** IdleStateB.

ERD/ERS has been studied widely as one of the brain activity markers for motor imagery tasks. Figure 5 demonstrates the grouped ERSP across 32 participants in the time-frequency (TF) plots on all electrodes and in the group-level 2-D scalp topographies during each stage of the sit-to-stand and stand-to-sit experiments (excluding the rest period). The ERSP estimates ERD/ERS from the entire duration of the trials relative to the baseline spectra from 4 to 30 Hz. All present ERSP values were significant (see Figure 5, ERD in blue, ERS in red) compared to the baseline (α = 0.05). There was a tendency to decrease the alpha-band power for the action observation stage in all sit-to-stand and stand-to-sit trials, indicating ERD mainly in the parietal and parieto-occipital regions. Only for the motor imagery stage, the ERD sustained toward the centro-parietal and central electrode sites was found. However, this ERD was not present for the idle state in all trials of both experiments. Furthermore, we observed a significant increase in the beta-band power, indicating ERS, in the motor imagery stage of sit-to-stand and stand-to-sit trials compared to the idle state.

The Fisher's criterion was applied to evaluate the extracted features from each participant and show the highest rank features, as reported in Table 1. The features most common to all participants for the sit-to-stand and stand-to-sit classification scenarios were the low-beta frequency band with the fifth spatial filter and the alpha frequency band with the sixth spatial filter, respectively. In both classification scenarios, the highest Fisher score values were 5.08 and 9.65 in the low-beta frequency band of participant ID P25. The lowest Fisher score values were 0.32 for the sit-to-stand classification scenario in the theta frequency band of participant ID P03 and 0.34 for the stand-to-sit classification scenario in the alpha frequency band of participant ID P13.

**Table 1:** Comparison of the highest-ranking features.

**Participant ID**	**Sit-to-stand**			**Stand-to-sit**		
	**Feature**		**Fisher score value**	**Feature**		**Fisher score value**
	**Rhythm**	**Spatial filter**		**Rhythm**	**Spatial filter**	
P01	Alpha	1	3.64	High Beta	2	2.03
P02	Low Beta	4	1.23	Mid Beta	1	0.52
P03	Theta	5	0.32	Alpha	4	0.40
P04	Low Beta	6	2.61	Alpha	6	2.01
P05	High Beta	6	1.65	High Beta	2	1.13
P06	Alpha	5	2.71	Alpha	4	1.98
P07	Mid Beta	2	1.31	High Beta	2	1.68
P08	Mid Beta	6	0.58	Low Beta	6	0.54
P09	Low Beta	5	2.32	Low Beta	5	1.93
P10	Alpha	5	3.97	Alpha	6	1.75
P11	High Beta	5	1.13	Alpha	6	1.70
P12	Mid Beta	2	0.57	Theta	1	0.88
P13	Alpha	6	0.67	Alpha	5	0.34
P14	Alpha	5	0.87	Alpha	1	1.00
P15	Alpha	1	0.79	Alpha	5	0.54
P16	Theta	1	3.04	Alpha	2	3.33
P17	Mid Beta	1	2.14	Mid Beta	1	1.61
P18	High Beta	6	0.71	Alpha	5	0.57
P19	Low Beta	6	0.84	Alpha	6	0.72
P20	Low Beta	1	1.04	Low Beta	2	2.45
P21	Theta	6	0.62	Mid Beta	6	0.55
P22	Low Beta	4	1.00	Theta	1	0.95
P23	Low Beta	5	4.82	High Beta	2	0.63
P24	Low Beta	1	0.90	High Beta	1	1.15
P25	Low Beta	5	5.08	Low Beta	6	9.65
P26	High Beta	2	1.30	Theta	2	0.99
P27	Low Beta	6	2.39	Alpha	5	1.63
P28	Alpha	6	0.72	Alpha	1	1.51
P29	Low Beta	5	3.10	Low Beta	5	2.16
P30	Low Beta	5	0.98	Alpha	6	0.80
P31	Low Beta	6	2.19	Alpha	6	1.24
P32	Theta	6	1.61	Alpha	3	1.05

Features were ranked using Fisher's separability criterion.

**Figure 6:**
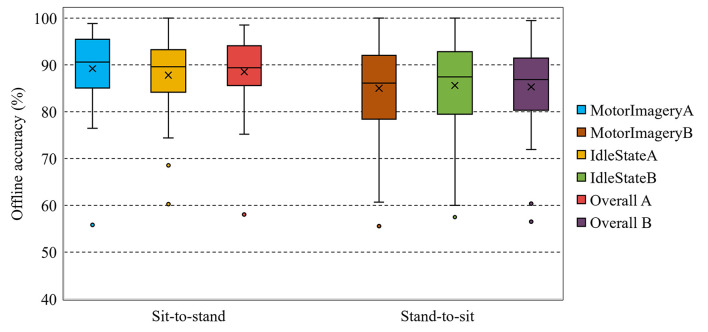
Classification accuracies (%) estimated with five-fold cross-validation across all participants for the motor imagery vs. idle state classes in the sit-to-stand and stand-to-sit offline experiments. × represents the mean. The Supplementary material section provides the result for each participant.

Box plots were used to present the distribution of the offline classification accuracy estimated with the five-fold cross-validation procedure across all participants (see Figure 6). In particular, the mean accuracies (denoted by ×) of the MotorImageryA, IdleStateA, and overall A classes were, respectively, 89.21, 87.81, and 88.51% in the sit-to-stand classification scenario. Likewise, the medians of the MotorImageryA, IdleStateA, and overall A classes were 90.58, 89.60, and 89.36%, respectively. The worst classifier performances were below 80% (2 women and 1 man), whereas 15 participants obtained classifier performances above 90% (7 women and 8 men). The best model performance was 98.49% and the worst was 58.02%.

In the stand-to-sit offline classification scenario, the mean accuracies of the MotorImageryB, IdleStateB, and overall B classes were, respectively, 84.99, 85.60, and 85.29%. Similarly, the medians of the MotorImageryB, IdleStateB, and overall B classes were 86.12, 87.43, and 86.83%, respectively. Additionally, 7 participants obtained classifier performances below 80% (4 women and 3 men) and 10 participants above 90% (6 women and 4 men). In this case, the best model performance was 99.44% and the worst was 56.51%.

In the permutation tests, the overall classification accuracies in 30 of 32 participants were statistically significant (*p* < 0.05, 1,000 random permutations) for the sit-to-stand and stand-to-sit classification scenarios. Only participant ID P03 presented *p*-values higher than 0.05 for both classification scenarios, which means not statistically significant. Likewise, for the stand-to-sit classification scenario, the *p*-values of participant ID P13 were higher than 0.05, not statistically significant, and indicate strong evidence for the null hypothesis. Altogether, the offline classification results showed the feasibility of recognizing the studied motor imagery tasks vs. idle state above empirical chance levels. The Supplementary material section provides the results for each participant.

**Figure 7:**
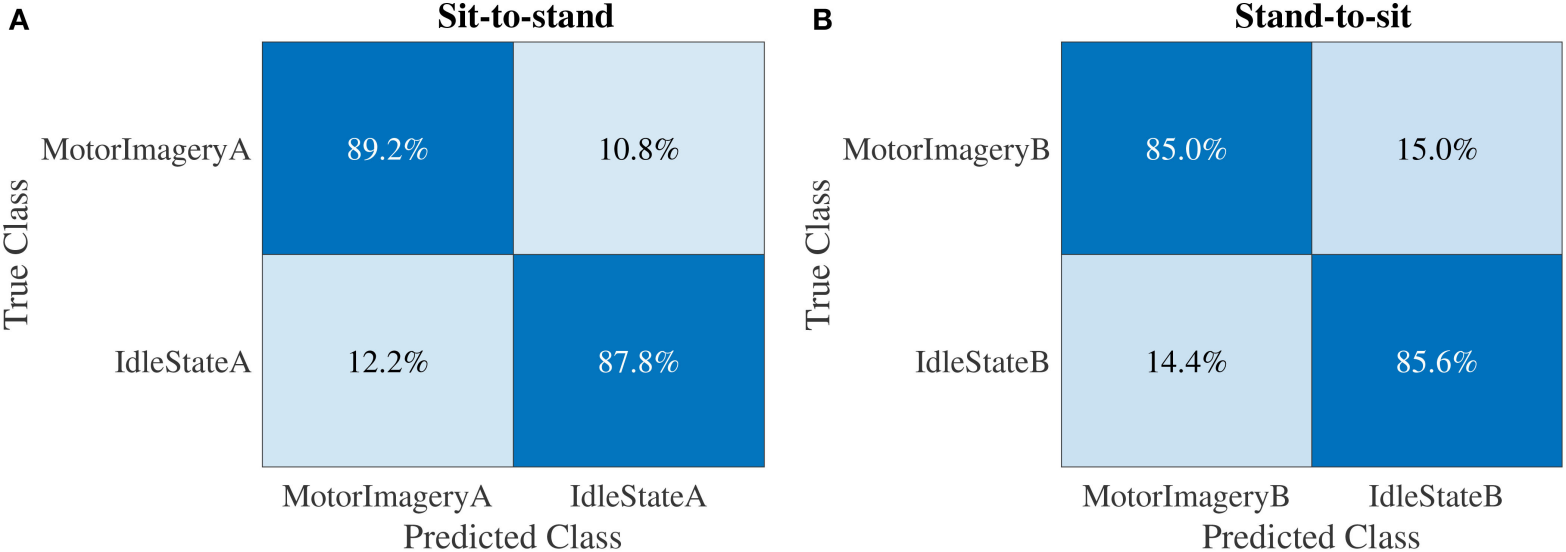
Confusion matrices obtained in the sit-to-stand **(A)** and stand-to-sit **(B)** offline classification scenarios for all participants.

Figure 7 shows the results of the confusion matrices obtained in the sit-to-stand and stand-to-sit classification scenarios for the MotorImagery vs. IdleState classes. Regarding the confusion matrix in Figure 7A, corresponding to the sit-to-stand scenario, the true positive rate (*TPR*), false negative rate (*FNR*), false positive rate (*FPR*), and true negative rate (*TNR*) were, respectively, 89.2, 10.8, 12.2, and 87.8%. As for the confusion matrix in Figure 7B, for the stand-to-sit scenario, the *TPR*, *FNR*, *FPR*, and *TNR* were 85.0, 15.0, 14.4, and 85.6%, respectively. Overall, the sit-to-stand and stand-to-sit offline classification scenarios showed comparable results and a relatively balanced performance among the different classes.

**Figure 8:**
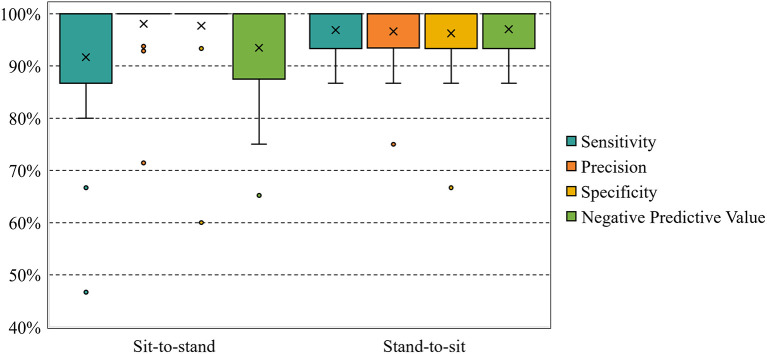
Across-all-participants distributions (%) of sensitivity, precision, specificity, and negative predictive value obtained in the sit-to-stand and stand-to-sit online experiments. ×represents the mean. The Supplementary material section provides the result for each participant.

The box plots in Figure 8 were used to show the characteristics of the sensitivity, precision, specificity, and negative predictive value calculated across all participants in the sit-to-stand and stand-to-sit online classification scenarios. In the sit-to-stand scenario, most of the characteristics had a mean (indicated by ×) of between 90 and 100% and a median of 100% for both the women and men groups. With respect to the stand-to-sit scenario, these characteristics had also a mean of between 90 and 100% and a median of 100%. These results indicate that models for the sit-to-stand scenario can discriminate between EEG epochs of MotorImagery vs. IdleState classes just as well as models for the stand-to-sit scenario.

**Figure 9:**
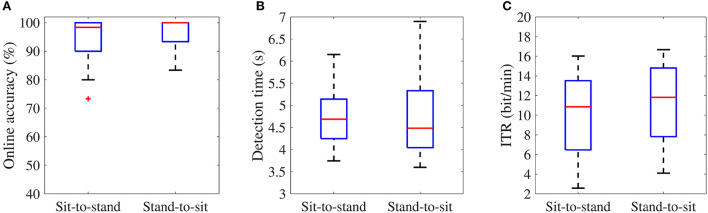
Box plots of the online accuracy **(A)**, task detection time **(B)**, and ITR values **(C)** of the BCI online experiments. The Supplementary material section provides the result for each participant.

Finally, the distribution of the online accuracy, detection time and ITR in the sit-to-stand and stand-to-sit online experiments are represented in Figure 9. The mean accuracies ± standard error of the sit-to-stand and stand-to-sit online experiments were 94.69 ± 1.29% and 96.56 ± 0.83%, respectively. The average detection times were 4.70 ± 0.11 s and 4.77 ± 0.16 s, and the mean ITRs were 10.12 ± 0.73 bit/min and 11.13 ± 0.72 bit/min for the sit-to-stand and stand-to-sit online experiments, respectively. The shortest detection times were 3.75 s in the sit-to-stand experiments and 3.60 s in the stand-to-sit experiments. The longest detection times were 6.15 s in the sit-to-stand experiments and 6.90 s in the stand-to-sit experiments. Likewise, the minimum ITRs were 2.57 bits per minute in the sit-to-stand experiments and 4.10 bits per minute in the stand-to-sit experiments. The maximum ITRs were 16.02 bits per minute in the sit-to-stand experiments and 16.68 bits per minute in the stand-to-sit experiments.

## 4. Discussion

In this study, we found that sitting and standing motor imagery tasks can be recognized online using an EEG-based BCI. From our findings, a high percentage of participants (above 80%) can control the motor imagery (MI)-based BCI for standing and sitting. In addition to expanding the participant sample size, using more EEG active electrodes, and improving signal processing, this study enhanced previous research by proposing a solution to the problem of online decoding of motor imagery electroencephalography (MI-EEG) signals for standing and sitting.

For the first time, decoding EEG rhythms offline and online during motor imagery tasks for standing and sitting had a satisfactory performance using a feasible BCI paradigm. Current BCI paradigms that consider the complexity of shifting from sitting to standing and vice versa are usually either based on left-hand and right-hand motor imagery tasks (Noda et al., 2012; Wang et al., 2018) or SSVEP signals (Kwak et al., 2017). These BCI paradigms have proven effective in transferring information from the brain to a computer. However, they are unnatural for the brain to interact with and thus require much more cognitive resources to act as traditional human-computer interfaces for the sit-to-stand and stand-to-sit transitions. For this reason, one of the main contributions of the proposed BCI paradigm is to provide a more natural interaction between the user and the interface, which is a current challenge in the design of BCI systems (Xu et al., 2021).

Previous studies have shown the effects of task complexity on ERD/ERS rhythms and the complexity of the sit-to-stand and stand-to-sit movements (Bulea et al., 2014; Singh et al., 2017; Mashat et al., 2019; Chaisaen et al., 2020). To overcome these limitations, we used information from the idle state, a neutral condition, to facilitate the classifier's recognition of distinctive characteristics of the related motor imagery task. ERD BCIs have pursued this approach with considerable success generating brain signals that are easier to categorize (see Figure 5). This BCI paradigm with a low cognitive load could explain why our study did not show the typical BCI inefficiency (Allison and Neuper, 2010; Edlinger et al., 2015; Liu et al., 2020; Xu et al., 2021). Additionally, the frequency bands and spatial filters of the extracted features reported in Table 1 could provide the most discriminated information.

The overall classification accuracies (see Figure 6), estimated with cross-validation in the offline classification scenarios, are similar to those reported in other related BCI literature (Wang et al., 2018; Chaisaen et al., 2020). For instance, in the offline analysis by Chaisaen et al. (2020), the classification of action observation (AO) and motor imagery (MI) provided the grand average accuracy ± standard error (SE) of 82.73 ± 2.54% in the stand-to-sit transition, which is lower compared to 85.29 ± 1.83% between the classification of motor imagery and idle state in this study. In the current study, the highest grand average accuracy ± SE was 88.51 ± 1.43% between the classification task of motor imagery and idle state in the sit-to-stand transition, compared to 76.14 ± 3.14% in the classification of AO and MI by Chaisaen et al. (2020). Furthermore, within the 60 min of training, 30 of 32 participants achieved an overall accuracy above chance level.

The confusion matrices (see Figure 7) showed that the trained classification models generated balanced results for the different classes. The overall accuracy results obtained in the classification models trained with the permutation testing method also confirm this situation empirically (see Supplementary material section). These results show that the classifiers were not highly biased toward any experimental task. Furthermore, sensitivity, precision, specificity, negative predictive value, and accuracy in the online phase are not measured by cross-validation as in offline experiments. Therefore, due to the heuristics described to detect one experimental task and compute the performance metrics in the online experiments, these metrics are not directly comparable between the online and offline phases.

The online classification results (see Figure 8) demonstrated the feasibility of the BCI to decode real-time EEG rhythms during the studied motor imagery tasks, whereas previous studies usually only presented classification accuracy. We also calculated sensitivity, precision, specificity, and negative predictive value to illustrate the online detection ability of the BCI to motor imagery-related potentials vs. idle state potentials. When considering all participants as a single group, the mean accuracies ± SE of the sit-to-stand and stand-to-sit online experiments were 94.69 ± 1.29% and 96.56 ± 0.83%, respectively, which are above the range of previous studies (Noda et al., 2012; Wang et al., 2018; Choi et al., 2020).

In both online classification scenarios (see Figure 9), the number of processed epochs and thresholds for motor imagery and idle state classes are customizable for each participant to improve online accuracy, detection time, and ITR of the MI-based BCI system. However, we used the same parameters for all participants, and it is essential to improve the system performance for participants who cannot achieve high detection rates. One potential strategy for improving system performance would be to modify the detection criteria in the third step of the online timeline. For instance, the classification of multiple sliding windows per trial provides a simple way to find a balance between the detection speed and the average accuracy of the system (Lee et al., 2019).

Both the offline (five-fold cross-validated data) and online (not cross-validated data) classification results demonstrated that the MI-based BCI could identify new observations of each class with high accuracy. Nevertheless, the results are similar to those reported in other MI-based BCI studies (Irimia et al., 2018; Choi et al., 2020; Gurve et al., 2020). These may be due to the high motivation of the research subjects. Additionally, the instructions provided during the subjects' training emphasized the differences between the studied motor imagery tasks with a high cognitive load vs. the no imaginary movement state (idle or rest state) with a low cognitive load to achieve a good performance. At the same time, the strategy applied to increase the online accuracy by single-trial classification using sliding windows allowed to reduce the classification errors but slowed down the detection time and ITR. Therefore, the choice of the parameters is crucial to keep a balance between the detection speed and the classification accuracy of the interface.

If we consider the response times of the present BCI system, it is not possible to implement an active fine control for a robotic device. However, by improving the response times of this new BCI, users could send commands to standing devices (like standing wheelchairs) to execute complete sit-to-stand or stand-to-sit transitions using the interface. For this reason, it is our view that the MI-based BCI system is suitable for the movements studied in this research.

One of the difficulties encountered in this study is the lack of an objective comparison between offline vs. online results. Ideally, the online classification performance should be calculated similarly to the values calculated using the cross-validation procedure of the offline phase. However, the crucial problem is to perform cross-validation with only a few observations in the online phase because it may lead to overfitting and poor generalization. Hence, cross-validation was necessary to evaluate the classification performance of the machine learning models in the offline phase. By contrast, in the online phase, the classification of multiple sliding windows per trial addressed the problem of single-trial misclassification and false positives in order to evaluate the online classification performance of the models (Mendoza-Montoya, 2017; Delijorge et al., 2020; Hernandez-Rojas et al., 2022).

The results suggest that a large population can control the EEG-based BCI and that high accuracy of above 90% can be achieved. Further research is required to establish whether people suffering from mobility impairments (who had previously been able to stand up and sit down before the impairments developed) could perform motor imagery tasks and operate the EEG-based BCI system for standing and sitting. Furthermore, more techniques for feature extraction and more machine learning models also are considered to extend the analyses. Classification techniques such as deep learning can be another alternative to analyze the problem studied here. Another interesting aspect would be to include more motor imagery tasks (e.g., three-class classification: imagining standing vs. imagining sitting vs. resting) that the system can interpret and test in more realistic environments. This system could represent the basis for modern interfaces' integration into future technologies (e.g., exoskeleton-based rehabilitation systems or brain-controlled standing wheelchairs) where the interface can be adapted to the user's specific disability.

## Data availability statement

The raw data supporting the conclusions of this article will be made available by the authors, without undue reservation.

## Ethics statement

The studies involving human participants were reviewed and approved by the Ethics Committee of the Universidad Antonio Nariño. The patients/participants provided their written informed consent to participate in this study.

## Author contributions

NT-G, AO-C, and AJ conceived and developed the presented idea. NT-G, OM-M, and JA participated in the design and implementation of the study. NT-G performed the experiments, conducted the research, and responsible for writing the manuscript. AO-C, AJ, OM-M, and JA supervised NT-G research's data analysis, methods, and findings. All authors contributed to the article and approved the submitted version.

## Funding

This research was funded by Ministerio de Ciencia, Tecnología e Innovación of Colombia-Minciencias/Colciencias, contract 594-2019, project 80740-594-2019, and in part by the University of Illinois Chicago and Tecnologico de Monterrey (UIC-TEC) Seed funding Program 2021-2022.

## Conflict of interest

The authors declare that the research was conducted in the absence of any commercial or financial relationships that could be construed as a potential conflict of interest.

## Publisher's note

All claims expressed in this article are solely those of the authors and do not necessarily represent those of their affiliated organizations, or those of the publisher, the editors and the reviewers. Any product that may be evaluated in this article, or claim that may be made by its manufacturer, is not guaranteed or endorsed by the publisher.

## References

[B1] Aggarwal S. Chugh N. (2019). Signal processing techniques for motor imagery brain computer interface: a review. Array 1–2, 1–12. 10.1016/j.array.2019.10000330459588

[B2] Ahn M. Cho H. Ahn S. Jun S. C. (2013). High theta and low alpha powers may be indicative of BCI-illiteracy in motor imagery. PLoS ONE 8, e80886. 10.1371/journal.pone.0080886 24278339 PMC3838377

[B3] Al-Fahoum A. S. Al-Fraihat A. A. (2014). Methods of EEG signal features extraction using linear analysis in frequency and time-frequency domains. ISRN Neurosci. 2014, 1–7. 10.1155/2014/730218 24967316 PMC4045570

[B4] Allison B. Z. Neuper C. (2010). “Could anyone use a BCI?” in Brain-Computer Interfaces: Applying our Minds to Human-Computer Interaction, Chapter 3, 1st Edn (London: Springer), 35–54.

[B5] Ang K. K. Chin Z. Y. Wang C. Guan C. Zhang H. (2012). Filter bank common spatial pattern algorithm on BCI competition IV datasets 2a and 2b. Front. Neurosci. 6, 39. 10.3389/fnins.2012.00039 PMC3314883 22479236

[B6] Asanza V. Peláez E. Loayza F. Lorente-Leyva L. L. Peluffo-Ordóñez D. H. (2022). Identification of lower-limb motor tasks via brain-computer interfaces: a topical overview. Sensors 22, 1–24. 10.3390/s22052028 35271175 PMC8914806

[B7] Association W. M. (2013). World medical association declaration of helsinki: ethical principles for medical research involving human subjects. JAMA 310, 2191–2194. 10.1001/jama.2013.281053 24141714

[B8] Berrar D. (2019). “Cross-validation,” in Encyclopedia of Bioinformatics and Computational Biology, Vol. 1 (Oxford: Academic Press Inc.), 542–545.

[B9] Blankertz B. Curio G. Müller K.-R. (2001). “Classifying single trial EEG: towards brain computer interfacing,” in Advances in Neural Information Processing Systems, Vol. 14 (Vancouver, BC: MIT Press), 157–164.

[B10] Blankertz B. Losch F. Krauledat M. Dornhege G. Curio G. Muller K.-R. (2008a). The berlin brain-computer interface: accurate performance from first-session in BCI-naive subjects. IEEE Trans. Biomed. Eng. 55, 2452–2462. 10.1109/TBME.2008.923152 18838371

[B11] Blankertz B. Tomioka R. Lemm S. Kawanabe M. Muller K.-R. (2008b). Optimizing spatial filters for robust EEG single-trial analysis. IEEE Signal Process Mag. 25, 41–56. 10.1109/MSP.2008.4408441

[B12] Bobrova E. V. Reshetnikova V. V. Frolov A. A. Gerasimenko Y. P. (2020). Use of imaginary lower limb movements to control brain-computer interface systems. Neurosci. Behav. Physiol. 50, 585–592. 10.1007/s11055-020-00940-z

[B13] Bulea T. C. Prasad S. Kilicarslan A. Contreras-Vidal J. L. (2014). Sitting and standing intention can be decoded from scalp EEG recorded prior to movement execution. Front. Neurosci. 8, 376. 10.3389/fnins.2014.00376 PMC4243562 25505377

[B14] Chaisaen R. Autthasan P. Mingchinda N. Leelaarporn P. Kunaseth N. Tammajarung S.. (2020). Decoding EEG rhythms during action observation, motor imagery, and execution for standing and sitting. IEEE Sens J. 20, 13776–13786. 10.1109/JSEN.2020.3005968

[B15] Chen W. Wang S. Zhang X. Yao L. Yue L. Qian B.. (2018). “EEG-based motion intention recognition via multi-task RNNs,” in Proceedings of the 2018 SIAM International Conference on Data Mining (SDM) (San Diego, CA: Society for Industrial and Applied Mathematics Publications), 279–287.

[B16] Choi J. Kim K. T. Jeong J. H. Kim L. Lee S. J. Kim H. (2020). Developing a motor imagery-based real-time asynchronous hybrid BCI controller for a lower-limb exoskeleton. Sensors 20, 1–15. 10.3390/s20247309 33352714 PMC7766128

[B17] Congedo M. Barachant A. Bhatia R. (2017). Riemannian geometry for EEG-based brain-computer interfaces; a primer and a review. Brain Comput. Interfaces 4, 155–174. 10.1080/2326263X.2017.1297192

[B18] Delijorge J. Mendoza-Montoya O. Gordillo J. L. Caraza R. Martinez H. R. Antelis J. M. (2020). Evaluation of a P300-based brain-machine interface for a robotic hand-orthosis control. Front. Neurosci. 14, 589659. 10.3389/fnins.2020.589659 PMC7729175 33328860

[B19] Delorme A. Makeig S. (2004). EEGLAB: an open source toolbox for analysis of single-trial EEG dynamics including independent component analysis. J. Neurosci. Methods 134, 9–21. 10.1016/j.jneumeth.2003.10.009 15102499

[B20] Edlinger G. Allison B. Z. Guger C. (2015). “How many people can use a BCI system?” in Clinical Systems Neuroscience, Chapter 3 (Tokyo: Springer), 33–66.

[B21] Fu R. Tian Y. Bao T. Meng Z. Shi P. (2019). Improvement motor imagery EEG classification based on regularized linear discriminant analysis. J. Med. Syst. 43, 1–13. 10.1007/s10916-019-1270-0 31062175

[B22] Gao Z. Wang Z. Ma C. Dang W. Zhang K. (2018). A Wavelet time-frequency representation based complex network method for characterizing brain activities underlying motor imagery signals. IEEE Access 6, 65796–65802. 10.1109/ACCESS.2018.2876547

[B23] George O. Smith R. Madiraju P. Yahyasoltani N. Ahamed S. I. (2021). “Motor imagery: a review of existing techniques, challenges and potentials,” in 2021 IEEE 45th Annual Computers, Software, and Applications Conference (COMPSAC) (Madrid: Institute of Electrical and Electronics Engineers Inc.), 1893–1899.

[B24] Good P. I. (2006). Resampling Methods: A Practical Guide to Data Analysis. Boston, MA: Birkhauser.

[B25] Graimann B. Pfurtscheller G. (2006). “Quantification and visualization of event-related changes in oscillatory brain activity in the time-frequency domain,” in Progress in Brain Research, Vol. 159, Chapter 6 (Amsterdam: Elsevier), 79–97. 10.1016/S0079-6123(06)59006-5 17071225

[B26] Gurve D. Delisle-Rodriguez D. Romero-Laiseca M. Cardoso V. Loterio F. Bastos T.. (2020). Subject-specific EEG channel selection using non-negative matrix factorization for lower-limb motor imagery recognition. J. Neural Eng. 17, 1–15. 10.1088/1741-2552/ab4dba 31614343

[B27] Hamedi M. Salleh S. H. Noor A. M. Mohammad-Rezazadeh I. (2014). “Neural network-based three-class motor imagery classification using time-domain features for BCI applications,” in 2014 IEEE Region 10 Symposium (Kuala Lumpur: Institute of Electrical and Electronics Engineers Inc.), 204–207.

[B28] He H. Wu D. (2018). “Spatial filtering for brain computer interfaces: a comparison between the common spatial pattern and its variant,” in 2018 IEEE International Conference on Signal Processing, Communications and Computing (ICSPCC) (Qingdao: Institute of Electrical and Electronics Engineers Inc.), 1–6.

[B29] He Y. Eguren D. Azorín J. M. Grossman R. G. Luu T. P. Contreras-Vidal J. L. (2018). Brain-machine interfaces for controlling lower-limb powered robotic systems. J. Neural Eng. 15, 1–15. 10.1088/1741-2552/aaa8c0 29345632

[B30] Hernandez-Rojas L. G. Cantillo-Negrete J. Mendoza-Montoya O. Carino-Escobar R. I. Leyva-Martinez I. Aguirre-Guemez A. V.. (2022). Brain-computer interface controlled functional electrical stimulation: evaluation with healthy subjects and spinal cord injury patients. IEEE Access 10, 46834–46852. 10.1109/ACCESS.2022.3170906

[B31] Irimia D. C. Ortner R. Poboroniuc M. S. Ignat B. E. Guger C. (2018). High classification accuracy of a motor imagery based brain-computer interface for stroke rehabilitation training. Front. Rob. AI 5, 130. 10.3389/frobt.2018.00130 33501008 PMC7805943

[B32] Kee C.-Y. Ponnambalam S. G. Loo C.-K. (2017). Binary and multi-class motor imagery using Renyi entropy for feature extraction. Neural Comput. Appl. 28, 2051–2062. 10.1007/s00521-016-2178-y

[B33] Kevric J. Subasi A. (2017). Comparison of signal decomposition methods in classification of EEG signals for motor-imagery BCI system. Biomed. Signal Process. Control 31, 398–406. 10.1016/j.bspc.2016.09.007

[B34] Kwak N.-S. Müller K.-R. Lee S.-W. (2017). A convolutional neural network for steady state visual evoked potential classification under ambulatory environment. PLoS ONE 12, e172578. 10.1371/journal.pone.0172578 28225827 PMC5321422

[B35] Ledoit O. Wolf M. (2004). A well-conditioned estimator for large-dimensional covariance matrices. J. Multivar. Anal. 88, 365–411. 10.1016/S0047-259X(03)00096-4

[B36] Lee M. H. Kwon O. Y. Kim Y. J. Kim H. K. Lee Y. E. Williamson J.. (2019). EEG dataset and OpenBMI toolbox for three BCI paradigms: an investigation into BCI illiteracy. Gigascience 8, 1–16. 10.1093/gigascience/giz002 30698704 PMC6501944

[B37] Liu T. Huang G. Jiang N. Yao L. Zhang Z. (2020). Reduce brain computer interface inefficiency by combining sensory motor rhythm and movement-related cortical potential features. J. Neural Eng. 17, 1–9. 10.1088/1741-2552/ab914d 32380494

[B38] Lotte F. Bougrain L. Cichocki A. Clerc M. Congedo M. Rakotomamonjy A.. (2018a). A review of classification algorithms for EEG-based brain-computer interfaces: a 10 year update. J. Neural Eng. 15, 1–28. 10.1088/1741-2552/aab2f2 29488902

[B39] Lotte F. Guan C. (2011). Regularizing common spatial patterns to improve BCI designs: unified theory and new algorithms. IEEE Trans. Biomed. Eng. 58, 355–362. 10.1109/TBME.2010.2082539 20889426

[B40] Lotte F. Nam C. S. Nijholt A. (2018b). “Introduction: evolution of brain-computer interfaces,” in Brain-Computer Interfaces Handbook: Technological and Theoretical Advance (Boca Raton, FL: Taylor & Francis; CRC Press), 1–11.

[B41] Makeig S. (1993). Auditory event-related dynamics of the EEG spectrum and effects of exposure to tones. Electroencephalogr. Clin. Neurophysiol. 86, 283–293. 10.1016/0013-4694(93)90110-H 7682932

[B42] Mao X. Li M. Li W. Niu L. Xian B. Zeng M.. (2017). Progress in EEG-based brain robot interaction systems xiaoqian. Comput. Intell. Neurosci. 2017, 1–25. 10.1155/2017/1742862 28484488 PMC5397651

[B43] Mashat M. E. M. Lin C.-T. Zhang D. (2019). Effects of task complexity on motor imagery-based brain-computer interface. IEEE Trans. Neural Syst. Rehabil. Eng. 27, 2178–2185. 10.1109/TNSRE.2019.2936987 31443036

[B44] Mendoza-Montoya O. (2017). Development of a Hybrid Brain-Computer Interface for Autonomous Systems (Doctoral dissertation). Freie Universität Berlin.

[B45] Naeem M. Brunner C. Pfurtscheller G. (2009). Dimensionality reduction and channel selection of motor imagery electroencephalographic data. Comput. Intell. Neurosci. 2009, 1–8. 10.1155/2009/537504 19536346 PMC2695957

[B46] Ng A. Y. Jordan M. I. (2001). “On Discriminative vs. Generative classifiers: a comparison of logistic regression and naive Bayes,” in Advances in Neural Information Processing Systems, Vol. 14 (Vancouver, BC: MIT Press), 841–848.

[B47] Noda T. Sugimoto N. Furukawa J. Sato M. A. Hyon S. H. Morimoto J. (2012). “Brain-controlled exoskeleton robot for BMI rehabilitation,” in 2012 12th IEEE-RAS International Conference on Humanoid Robots (Humanoids 2012) (Osaka: Institute of Electrical and Electronics Engineers Inc.), 21–27.

[B48] Oikonomou V. P. Georgiadis K. Liaros G. Nikolopoulos S. Kompatsiaris I. (2017). “A comparison study on EEG signal processing techniques using motor imagery EEG data,” in 2017 IEEE 30th International Symposium on Computer-Based Medical Systems (CBMS) (Thessaloniki: Institute of Electrical and Electronics Engineers Inc.), 781–786.

[B49] Ortiz M. Iáñez E. Contreras-Vidal J. L. Azorín J. M. (2020). Analysis of the EEG rhythms based on the empirical mode decomposition during motor imagery when using a lower-limb exoskeleton. A case study. Front. Neurorob. 14, 48. 10.3389/fnbot.2020.00048 PMC7482655 32973481

[B50] Ortner R. Scharinger J. Lechner A. Guger C. (2015). “How many people can control a motor imagery based BCI using common spatial patterns?” in 2015 7th International IEEE/EMBS Conference on Neural Engineering (NER) (Montpellier: Institute of Electrical and Electronics Engineers Inc.), 202–205.

[B51] Padfield N. Zabalza J. Zhao H. Masero V. Ren J. (2019). EEG-based brain-computer interfaces using motor-imagery: techniques and challenges. Sensors 19, 1–34. 10.3390/s19061423 30909489 PMC6471241

[B52] Pfurtscheller G. Lopes da Silva F. (1999). Event-related EEG/MEG synchronization and desynchronization: basic principles. Clin. Neurophysiol. 110, 1842–1857. 10.1016/S1388-2457(99)00141-8 10576479

[B53] Podder P. Mehedi Hasan M. Rafiqul Islam M. Sayeed M. (2014). Design and implementation of butterworth, chebyshev-I and elliptic filter for speech signal analysis. Int. J. Comput. Appl. 98, 12–18. 10.5120/17195-7390

[B54] Rejer I. Górski P. (2018). “EEG classification for MI-BCI with independent component analysis,” in Proceedings of the 10th International Conference on Computer Recognition Systems CORES 2017, Vol. 578 (Polanica Zdroj: Springer International Publishing AG), 393–402.

[B55] Rodríguez-Bermúdez García-Laencina,Rodríguez-Bermúdez G. García-Laencina P. J. (2012). Automatic and adaptive classification of electroencephalographic signals for brain computer interfaces. J. Med. Syst. 36, 51–63. 10.1007/s10916-012-9893-4 23117792

[B56] Rodríguez-Ugarte M. Iáñez E. Ortíz M. Azorín J. M. (2017). Personalized offline and pseudo-online BCI models to detect pedaling intent. Front. Neuroinform. 11, 45. 10.3389/fninf.2017.00045 PMC5504298 28744212

[B57] Saha S. Baumert M. (2020). Intra- and inter-subject variability in EEG-based sensorimotor brain computer interface: a review. Front. Comput. Neurosci. 13, 87. 10.3389/fncom.2019.00087 PMC6985367 32038208

[B58] Samuel O. W. Geng Y. Li X. Li G. (2017). Towards efficient decoding of multiple classes of motor imagery limb movements based on EEG spectral and time domain descriptors. J. Med. Syst. 41, 1–13. 10.1007/s10916-017-0843-z 29080913

[B59] Singh A. Hussain A. A. Lal S. Guesgen H. W. (2021). A comprehensive review on critical issues and possible solutions of motor imagery based electroencephalography brain-computer interface. Sensors 21, 1–35. 10.3390/s21062173 33804611 PMC8003721

[B60] Singh B. Wagatsuma H. Natsume K. (2017). The detection of the rise to stand movements using bereitschaftspotential from scalp electroencephalography (EEG). SICE J. Control Meas. Syst. Integrat. 10, 149–155. 10.9746/jcmsi.10.149

[B61] Syan C. S. Harnarinesingh R. E. (2010). Comparison of pre-processing and classification techniques for single-trial and multi-trial P300-based brain computer interfaces. Am. J. Appl. Sci. 7, 1219–1225. 10.3844/ajassp.2010.1219.1225

[B62] Thompson M. C. (2018). Critiquing the concept of BCI illiteracy. Sci. Eng. Ethics 25, 1217–1233. 10.1007/s11948-018-0061-1 30117107

[B63] Wang C. Wu X. Wang Z. Ma Y. (2018). Implementation of a brain-computer interface on a lower-limb exoskeleton. IEEE Access 6, 38524–38534. 10.1109/ACCESS.2018.2853628

[B64] Wolpaw J. R. Birbaumer N. McFarland D. J. Pfurtscheller G. Vaughan T. M. (2002). Brain-computer interfaces for communication and control. Clin. Neurophysiol. 113, 767–791. 10.1016/S1388-2457(02)00057-3 12048038

[B65] Xu M. He F. Jung T. P. Gu X. Ming D. (2021). Current Challenges for the practical application of electroencephalography-based brain-computer interfaces. Engineering 7, 1710–1712. 10.1016/j.eng.2021.09.011

[B66] Xu R. Allison B. Z. Ortner R. Irimia D. C. Espinosa A. Lechner A.. (2017). “How many EEG channels are optimal for a motor imagery based BCI for stroke rehabilitation?” in Converging Clinical and Engineering Research on Neurorehabilitation II, Vol. 15 (Segovia: Springer International Publishing), 1109–1113.

[B67] Yu T. Xiao J. Wang F. Zhang R. Gu Z. Cichocki A.. (2015). Enhanced motor imagery training using a hybrid BCI with feedback. IEEE Trans. Biomed. Eng. 62, 1706–1717. 10.1109/TBME.2015.2402283 25680205

[B68] Yuan H. He B. (2014). Brain-computer interfaces using sensorimotor rhythms: current state and future perspectives. IEEE Trans. Biomed. Eng. 61, 1425–1435. 10.1109/TBME.2014.2312397 24759276 PMC4082720

[B69] Zhang Z. (2019). “Spectral and time-frequency analysis,” in EEG Signal Processing and Feature Extraction, Chapter 6 (Singapore: Springer Singapore), 89–116.

[B70] Zhou Z.-X. Ming D. Wan B.-K. Cheng L.-L. (2007). “Event-related EEG-changes during attempted standing up task,” in 2007 Joint Meeting of the 6th International Symposium on Noninvasive Functional Source Imaging of the Brain and Heart and the International Conference on Functional Biomedical Imaging (Hangzhou: Institute of Electrical and Electronics Engineers Inc.), 66–69.

