# Supplementary material for: Decoding EEG rhythms offline and online during motor imagery for standing and sitting based on a brain-computer interface
Source: Front Neuroinform. 2022 Sep 2;16:961089. doi: 10.3389/fninf.2022.961089 (PMC9481272; doi:10.3389/fninf.2022.961089)
Supplement: Supplementary file 1 [file Data_Sheet_1.PDF]

frontiers

Supplementary Material

Table S1. Classification accuracies (in %) ± standard error (SE) estimated with five-fold cross-validation for each participant for the motor imagery vs. idle state classes in the sit-to-stand and stand-to-sit offline experiments. Bold and * represents non-statistically significant differences between the means of the Overall and Overall random distributions ($p > 0.05$, 1,000 random permutations).

Participant ID	Sit-to-stand					Stand-to-sit				
	**MotorImageryA**	**IdleStateA**	**Overall A**	**Overall A random**	p**-value**	**MotorImageryB**	**IdleStateB**	**Overall B**	**Overall B random**	p**-value**
**P01**	98.39	98.59	98.49	49.72	0.0002	98.82	97.67	98.25	50.03	0.0002
**P02**	96.51	89.53	93.02	49.97	0.0005	79.76	77.65	78.70	49.88	0.0021
**P03**	55.81	60.23	58.02	49.90	**0.1731***	60.67	60.00	60.34	50.09	**0.0949***
**P04**	94.38	92.50	93.44	49.99	0.0004	86.36	84.27	85.32	8.32	0.0015
**P05**	95.56	91.76	93.66	50.11	0.0004	92.05	90.80	91.43	49.82	0.0005
**P06**	89.77	87.78	88.78	49.86	0.0006	90.00	87.36	88.68	50.13	0.0007
**P07**	83.72	89.77	86.75	49.93	0.0007	82.95	82.95	82.95	49.93	0.0013
**P08**	85.06	79.78	82.42	49.72	0.0017	82.22	78.89	80.56	50.00	0.0018
**P09**	93.26	97.75	95.51	50.00	0.0003	97.65	98.84	98.24	50.14	0.0003
**P10**	97.78	96.05	96.92	49.78	0.0003	83.72	86.05	84.88	49.82	0.0009
**P11**	92.05	82.56	87.30	50.14	0.0008	94.32	95.24	94.78	49.63	0.0003
**P12**	80.90	84.27	82.58	49.72	0.0035	72.73	74.44	73.59	50.05	0.0079
**P13**	81.82	68.54	75.18	49.92	0.0036	55.56	57.47	56.51	50.14	**0.2422***
**P14**	92.86	85.37	89.11	49.90	0.0009	85.88	93.10	89.49	50.23	0.0007
**P15**	82.22	88.76	85.49	49.82	0.0012	77.53	82.95	80.24	50.03	0.0041
**P16**	95.56	100.00	97.78	49.97	0.0003	95.40	100.00	97.70	49.98	0.0003
**P17**	86.21	93.02	89.62	49.91	0.0005	88.24	90.28	89.26	50.07	0.0005
**P18**	83.72	90.80	87.26	50.21	0.0018	74.39	73.56	73.98	49.95	0.0170
**P19**	90.36	79.07	84.72	49.94	0.0010	78.21	87.50	82.85	49.93	0.0012
**P20**	87.50	89.33	88.42	49.77	0.0006	98.85	100.00	99.43	49.93	0.0002
**P21**	76.47	74.39	75.43	49.79	0.0039	77.11	86.52	81.81	50.13	0.0016
**P22**	91.67	89.02	90.35	50.01	0.0006	87.78	92.05	89.91	50.15	0.0010
**P23**	96.59	93.33	94.96	49.74	0.0003	76.40	67.42	71.91	50.16	0.0110
**P24**	87.78	87.64	87.71	49.85	0.0008	87.78	88.89	88.33	49.73	0.0006
**P25**	97.65	94.19	95.92	50.04	0.0003	100.00	98.89	99.44	50.32	0.0002
**P26**	94.81	92.22	93.51	49.73	0.0003	92.05	90.91	91.48	49.93	0.0005
**P27**	88.64	94.32	91.48	50.19	0.0005	92.05	88.76	90.40	49.82	0.0005
**P28**	85.06	78.82	81.94	49.68	0.0016	85.56	93.26	89.41	50.01	0.0006
**P29**	95.24	96.63	95.93	49.94	0.0003	96.59	92.05	94.32	49.84	0.0004
**P30**	87.78	84.09	85.93	50.02	0.0009	79.07	78.41	78.74	50.19	0.0022
**P31**	98.84	89.66	94.25	49.52	0.0004	87.36	81.93	84.64	50.01	0.0011
**P32**	90.80	90.00	90.40	49.92	0.0006	82.56	81.11	81.83	50.17	0.0020
**Mean**	89.21	87.81	88.51	49.90	0.0063	84.99	85.60	85.29	50.01	0.0125
**± SE**	± 1.49	± 1.53	± 1.43	± 0.03	± 0.0054	± 1.84	± 1.90	± 1.83	± 0.03	± 0.0080

Table S2. Sensitivity (or TPR), precision (or PPV), specificity (or TNR), and negative predictive value (NPV) in percentage ± standard error (SE) obtained for each participant in the sit-to-stand and stand-to-sit online experiments.

Participant ID	Sit-to-stand				Stand-to-sit			
	Sensitivity	Precision	Specificity	NPV	Sensitivity	Precision	Specificity	NPV
P01	46.67	100.00	100.00	65.22	93.33	93.33	93.33	93.33
P02	100.00	100.00	100.00	100.00	93.33	87.50	86.67	92.86
P03	86.67	100.00	100.00	88.24	100.00	75.00	66.67	100.00
P04	100.00	100.00	100.00	100.00	100.00	100.00	100.00	100.00
P05	100.00	100.00	100.00	100.00	100.00	100.00	100.00	100.00
P06	100.00	100.00	100.00	100.00	100.00	93.75	93.33	100.00
P07	100.00	100.00	100.00	100.00	100.00	100.00	100.00	100.00
P08	100.00	93.75	93.33	100.00	100.00	88.24	86.67	100.00
P09	100.00	100.00	100.00	100.00	100.00	100.00	100.00	100.00
P10	80.00	100.00	100.00	83.33	93.33	100.00	100.00	93.75
P11	80.00	100.00	100.00	83.33	100.00	100.00	100.00	100.00
P12	100.00	93.75	93.33	100.00	100.00	100.00	100.00	100.00
P13	66.67	100.00	100.00	75.00	86.67	100.00	100.00	88.24
P14	86.67	92.86	93.33	87.50	86.67	86.67	86.67	86.67
P15	100.00	100.00	100.00	100.00	86.67	100.00	100.00	88.24
P16	66.67	100.00	100.00	75.00	100.00	100.00	100.00	100.00
P17	100.00	100.00	100.00	100.00	100.00	93.75	93.33	100.00
P18	66.67	100.00	100.00	75.00	93.33	87.50	86.67	92.86
P19	80.00	100.00	100.00	83.33	93.33	93.33	93.33	93.33
P20	100.00	93.75	93.33	100.00	100.00	100.00	100.00	100.00
P21	93.33	100.00	100.00	93.75	100.00	100.00	100.00	100.00
P22	100.00	100.00	100.00	100.00	100.00	100.00	100.00	100.00
P23	100.00	100.00	100.00	100.00	100.00	100.00	100.00	100.00
P24	100.00	71.43	60.00	100.00	100.00	100.00	100.00	100.00
P25	100.00	100.00	100.00	100.00	100.00	100.00	100.00	100.00
P26	100.00	100.00	100.00	100.00	100.00	100.00	100.00	100.00
P27	100.00	100.00	100.00	100.00	100.00	100.00	100.00	100.00
P28	86.67	92.86	93.33	87.50	100.00	100.00	100.00	100.00
P29	100.00	100.00	100.00	100.00	100.00	100.00	100.00	100.00
P30	100.00	100.00	100.00	100.00	86.67	92.86	93.33	87.50
P31	100.00	100.00	100.00	100.00	86.67	100.00	100.00	88.24
P32	93.33	100.00	100.00	93.75	100.00	100.00	100.00	100.00
Mean	91.67	98.07	97.71	93.47	96.88	96.62	96.25	97.03
± SE	± 2.41	± 0.96	± 1.29	± 1.76	± 0.90	± 1.06	± 1.27	± 0.84

Table S3. Box plots of the online accuracy, task detection time, and ITR values ± standard error (SE) of the BCI online experiments for each participant.

Participant ID	Accuracy (%)		Average detection time (s)		ITR (bit/min)	
	Sit-to-stand	Stand-to-sit	Sit-to-stand	Stand-to-sit	Sit-to-stand	Stand-to-sit
**P01**	73.33	93.33	3.81	6.90	2.57	5.62
**P02**	100.00	90.00	4.17	5.74	14.39	5.55
**P03**	93.33	83.33	6.15	5.12	6.31	4.10
**P04**	100.00	100.00	4.67	5.25	12.84	11.42
**P05**	100.00	100.00	4.67	4.03	12.84	14.90
**P06**	100.00	96.67	5.11	3.88	11.74	12.21
**P07**	100.00	100.00	5.56	3.74	10.79	16.06
**P08**	96.67	93.33	5.53	4.26	8.57	9.10
**P09**	100.00	100.00	4.42	4.10	13.57	14.65
**P10**	90.00	96.67	4.70	5.32	6.78	8.90
**P11**	90.00	100.00	5.54	5.27	5.75	11.38
**P12**	96.67	100.00	4.33	4.46	10.93	13.45
**P13**	83.33	93.33	5.39	6.90	3.89	5.62
**P14**	90.00	86.67	5.83	5.86	5.47	4.44
**P15**	100.00	93.33	5.25	4.63	11.43	8.38
**P16**	83.33	100.00	4.06	3.86	5.18	15.54
**P17**	100.00	96.67	5.14	5.11	11.68	9.27
**P18**	83.33	90.00	4.72	6.56	4.44	4.86
**P19**	90.00	93.33	4.50	5.34	7.08	7.26
**P20**	96.67	100.00	4.93	4.05	9.60	14.82
**P21**	96.67	100.00	5.14	4.53	9.20	13.23
**P22**	100.00	100.00	4.72	4.47	12.71	13.43
**P23**	100.00	100.00	3.77	4.26	15.92	14.08
**P24**	80.00	100.00	4.84	4.29	3.45	13.98
**P25**	100.00	100.00	3.84	3.60	15.61	16.68
**P26**	100.00	100.00	4.32	4.04	13.88	14.85
**P27**	100.00	100.00	3.75	4.06	16.02	14.77
**P28**	90.00	100.00	4.81	3.89	6.62	15.42
**P29**	100.00	100.00	3.82	3.62	15.70	16.58
**P30**	100.00	90.00	4.46	5.58	13.46	5.71
**P31**	100.00	93.33	4.03	4.50	14.88	8.62
**P32**	96.67	100.00	4.44	5.39	10.67	11.13
**Mean**	94.69	96.56	4.70	4.77	10.12	11.13
**± SE**	± 1.29	± 0.83	± 0.11	± 0.16	± 0.73	± 0.72
